# The burden, support and needs of primary family caregivers of people experiencing schizophrenia in Beijing communities: a qualitative study

**DOI:** 10.1186/s12888-019-2052-4

**Published:** 2019-02-20

**Authors:** Lifen Chen, Yali Zhao, Juan Tang, Guanghui Jin, Yanli Liu, Xuexue Zhao, Chao Chen, Xiaoqin Lu

**Affiliations:** 1 Department of Education, Xuanwu Hospital, Capital Medical University, Beijing, China; 2 School of General Practice and Continuing Education, Capital Medical University, No. 10, Xitoutiao, You An Men Wai, Fengtai District, Beijing, 100069 China; 3 Hong Kong International Medical Clinic, Beijing, China

**Keywords:** Perspective, Family caregiver, Schizophrenia, Community

## Abstract

**Background:**

Family caregivers play crucial roles in taking care of people experiencing schizophrenia in the community. The burdens on and needs of caregivers of these patients should be emphasized. This study aimed to explore the perspective of family caregivers of people experiencing schizophrenia in the communities of Beijing in terms of the burdens of care and the acquisition and further need for support in order to provide guidance to health care providers regarding how to target therapeutic interventions for families of individuals experiencing schizophrenia and to provide recommendations for policy makers to tailor countermeasures and services.

**Methods:**

A total of 20 family caregivers of schizophrenia patients were enrolled in our study. A face-to-face and semi-structured in-depth qualitative interview study was conducted to explore the caregivers’ perspective on the burden on caregivers, support and further needs. This study was conducted in the community health service centres where the family caregivers regularly visit. The study was carried out according to good ethical practices, data analysis and reporting guidelines.

**Results:**

Most participants reported that they were suffering from heavy life burdens and had negative experiences with respect to obtaining social support, and they emphasized that they would require more support. Economic and daily housework burdens, limited social communication, and psychological stresses were the principal burdens. Support including financial, medical and information and educational support did not satisfy the needs of the caregivers and their patients. More financial support, respect, and rehabilitation institutions were reported to be needs of the caregivers.

**Conclusions:**

Family caregivers of people experiencing schizophrenia suffer from heavy physical and psychological burdens; however, the current support provided is insufficient. More services and better public attitudes should be considered for people experiencing schizophrenia and their caregivers.

## Background

Schizophrenia is a severe, persistent and disabling chronic mental disorder affecting more than 21 million people worldwide [1] and has a high disease burden [2]. A systematic literature review estimated that the prevalence of schizophrenia is 0.48% globally [3]. The lifetime prevalence of schizophrenia is 0.54% in mainland China [4] and 0.49% in Beijing [5]. There are 7.16 million people who have suffered from schizophrenia during their lifetime in China [6]. A study implemented in four provinces in China indicated that among individuals with a diagnosable mental illness, 8% had sought professional help at some time, and 5% had seen a mental health professional [7]. Based on the World Health Organization’s (WHO’s) Mental Health Action Plan 2013–2020, people experiencing schizophrenia are usually transferred from psychiatric institutional care to community-based care when their conditions are stable. Family members are expected to become the most important caregivers for people experiencing schizophrenia in the community context in China [8]. Schizophrenia has a significantly negative impact on both patients and their families [9]. It is evident by a systematic review that 4.9–10.1% of schizophrenic patients are at risk of suicide during their lifetime [10]. Meanwhile, family caregivers of people experiencing schizophrenia experience significant stresses and have a high level of burden [11]. These huge burdens include physical discomfort, disturbed routine habits, tension, violence, chronic sorrow, enormous stigma, role changes, social withdrawal, and financial/career difficulties, usually with a lack of resources and support [12–15]. Heavy burdens and inadequate support might cause caregivers to become unable to undertake the responsibilities of caring for these patients [11]. Understanding such burdens is important for the development of efficient interventions and the rationalization of health-care resource allocation [5]. Many instruments have been developed to assess the burden on caregivers of people experiencing schizophrenia [16]. Family caregiver burden is a global issue [17]. Social support and professional support are essential solutions to alleviate the burdens of caregivers dealing with people experiencing schizophrenia [18]. The Social Network Questionnaire (SNQ), which includes social contacts, affective support, instrument support, and supportive relationships, was developed [19] and validated [20] to evaluate caregiver social support. Ribe et al. [18] developed and validated a scale comprising seven questions to evaluate the professional support received by caregivers. Many programmes have been implemented to provide social support or professional support for people experiencing schizophrenia and their caregivers. The “Family-to-Family Support Program” [21], psychoeducation programmes [22], and mutual support groups for family caregivers [23] have generated positive outcomes for patients and their family caregivers.

In China, people who are experiencing severe mental diseases are treated in psychiatric hospitals. When their conditions are stable, they are cared for by their family at home and are followed-up and managed at community health service centres (CHSCs) [24].

In 2004, the Chinese government launched the “Central Government Support for the Local Management and Treatment of Severe Mental Illnesses Project”, the largest model project in mental health in China. By the end of 2010, a total of 200,000 follow-up visits were completed among 280,000 registered people with severe mental illnesses; free medication and treatment were provided 94,000 and 2400 times, respectively [25].

In 2015, the Office of the State Council of the PRC released the “National Mental Health Work Plan (2015-2020)” [26], which put forward the next development plan including increasing the publicity of mental health, strengthening the prevention and treatment of severe mental disorders, improving the mental health service network, training more mental health professionals, decreasing the societal and economic costs of disruptive behaviours by individuals with mental illness, and standardizing management of mental health service.

In Beijing, many policies and measures have been implemented to support disabled people, including people experiencing schizophrenia. These measures focused on social support, economic support, medical support, educational support and environmental support. For social support, patients can participate in vocational rehabilitation programmes and employment training [27], and they are provided basic medical and social insurance [28]. For economic support, living allowances for patients and subsidies for caregivers are provided [29, 30]. For medical support, people experiencing schizophrenia can receive free psychotropic medications and a free annual physical examination at CHSCs [31]. For educational support, the children of people experiencing schizophrenia are provided educational subsidies for schools [32]. For environmental support, the patient himself or his family can receive welfare support for improving living conditions [33].

In mainland China, studies of the burdens and supports for caregivers of people experiencing schizophrenia have been relatively few in number. This qualitative study aimed to explore the views of family caregivers of people experiencing schizophrenia in terms of burdens of care and the acquisition of and further need for support in order to guide health care providers regarding how to target therapeutic interventions for families of individuals experiencing schizophrenia and to provide recommendations to policy-makers to tailor countermeasures and services.

## Methods

### Study design

A qualitative exploratory study among family caregivers of people experiencing schizophrenia was conducted using face-to-face and semi-structured in-depth interviews. After being pilot-tested, the interview questions were as follows: (1) demographic characteristics of patients and caregivers; (2) “what difficulties have you encountered when caring for patients?”; (3) “what support or resources have you obtained?”; and (4) “what further support do you need in order to provide better care for the patients?”

### Participants

Twenty-three family caregivers of people experiencing schizophrenia were enrolled by purposive sampling from 6 communities in Beijing; however, three caregivers refused to participate in the interview without giving any reasons. Twenty caregivers were eventually interviewed. Written consent was obtained from the participants with full explanation of the purpose and procedure of the study. Confidentiality of data and personal information was assured to the participants.

The inclusion and exclusion criteria of the participants were listed. Inclusion criteria included (1) the patient was diagnosed with schizophrenia by a psychiatrist according to ICD-10 schizophrenia criteria; (2) the patient was rehabilitated or convalesced and registered and managed by doctors in CHSCs for more than one month; (3) the caregiver had cared for the patients for more than one year; (4) the caregiver was over 18 years old; (5) the caregiver was free from any mental disorders before the patients was diagnosed with schizophrenia; (6) the caregiver had no other severe physical diseases; and (7) the caregiver agreed to participate in the study. Exclusion criteria were (1) the caregiver suffered from mental illness; (2) the caregiver had difficulty with language or could not cooperate with the investigation; and (3) the person experiencing schizophrenia was not completely registered in the community.

The average age of the caregivers (C1–20) was 66.33 ± 11.39 years, while the average age of the people experiencing schizophrenia was 48.07 ± 10.65 years. The demographics of the patients and the caregivers are described in Table 1 and Table 2, respectively.

**Table 1:** Demographic characteristics of the people experiencing schizophrenia (*n* = 20)

Demographic characteristic		No. of Patients
Gender	Male	10
	Female	10
Education level	University	8
	Secondary school	6
	Primary school or below	6
Marital status	Married	6
	Single	10
	Divorced and Widowed	4
Work before illness	Yes	12
	No	8
Work during illness	Yes	5
	No	15
Hospitalization in the previous year	Yes	3
	No	17
Medical payment	The fundamental medical insurance system of urban employee	8
	The new basic medical insurance for urban residents	11
	Other	1
Regular medication	Yes	17
	No	3
Disease status^a^	Stable phase	8
	Relatively stable phase	9
	Unstable phase	3

^a^ [24]:Danger assessment: for all patients, comprising 6 grades

Grade 0: no behaviour in Grade 1–5

Grade 1: verbal threat, screaming, without violent behaviour

Grade 2: violent behaviour against properties at home but can be persuaded to stop

Grade 3: obvious violent behaviour at all places against properties, cannot be persuaded to stop

Grade 4: continuous violent behaviour at all places against properties or people, including hurting oneself and suicide, cannot be persuaded to stop

Grade 5: Any armed violent behaviour against people or setting fire or explosion at home or public places

Stable patients: Grade 0 in danger assessment, with self-awareness and normal or good social function, without mental symptoms and severe adverse medication effect, without severe physical disease or with stable physical disease

Relatively stable patients: Grade 1–2 in danger assessment or having problem in at least 1 aspect among mental symptoms, self-awareness, and social function

Unstable patients: Grade 3–5 in danger assessment or obvious mental symptoms without self-awareness or severe adverse medication effects or severe physical diseases

**Table 2:** Demographic characteristics of the caregiver interviewees (*n* = 20)

Demographic characteristic		No. of Caregivers
Gender	Male	9
	Female	11
Educational level	University	8
	Secondary school	10
	Primary school or below	2
Occupation	Employed	3
	Unemployed	2
	Retired	14
	Other	1
Marital status	Married	15
	Widowed/Divorced/Never married	5
Relationship with the patient	Parent	11
	Spouse	5
	Child	3
	Sibling	1
Religion	Yes	3
	No	17
Duration of caring experience	Less than 5 years	1
	5–10 years	3
	More than 10 years	16
Medical payment	The fundamental medical insurance system of urban employee	16
	The new basic medical insurance for urban residents	2
	Free medical service	2
Number of family members	Less than 3	5
	3	11
	More than 3	4

### Data collection

In-depth interviews lasting 60–90 min were conducted to identify the burden status, the support experienced by the caregivers and their needs for support services, from January to March 2016. Twenty caregivers were interviewed in quiet meeting rooms at 6 general practices. The interviewer from the research team was experienced in qualitative research and had engaged in community management research of chronic diseases including schizophrenia for ten years. She directed the entire process of the interviews. In the interviews, two team members familiar with qualitative and social research experiences took notes for ten interviews each, in addition to tape recording the sessions. The point of information saturation was reached at the twentieth interview. No one else was present besides the participants and 2 researchers. To standardize the interviewing process, the members of the research team received pre-study training in qualitative interview and analysis organized by the researchers. Regular meetings were held to control the data quality. The interviewers did not contact the participants before the study. Before the interview, the interviewer introduced the reason and value of the study to the participants. The procedures of the interview were as follows: (1) the interviewees who would like to participate in the study were contacted by the GPs at the CHSCs; six GPs from six communities supported our research and were willing to help us recruit the interviewees. (2) After informed consent was obtained, the interviewer arranged a face-to-face interview alone with each caregiver for approximately 30 min. (3) The sessions were recorded and transcribed verbatim; the true names of the interviewees were replaced by codes; and the privacy of the patients was well-preserved. (4) Interview records were independently extracted by two researchers, and differences in data abstraction were resolved by consensus.

### Data analysis

Responses were audio-recorded and documented in writing simultaneously. The raw data were transferred, coded, and categorized by two team researchers who were PhD degree candidates in general practice. Then, themes were extracted from the interviews for analysis. Analysis and interpretation were performed, and consensus was reached in research team meetings. The research team was a multidisciplinary team, which is critical to the depth of understanding, the design of study and the validity of the results.

In the analysis process, Colaizzi’s 7-step analysis method of phenomenological data [34] was used to (1) read the interview records carefully; (2) extract important and meaningful statements; (3) encode recurring and meaningful content; (4) collect encoded views; (5) write down detailed and exhaustive descriptions; (6) distinguish similar views and sublimate theme concepts; and (7) return findings to the participants to verify ambiguous information. The research team members then reconstructed the data in accordance with the established order and theme and achieved consensus based on discussion among team members.

## Results

Three dominant themes emerged from the insights of the caregivers: burden of caregivers, support, and further needs.

### Burden of caregivers

Three main dimensions converged in terms of the burden of caregivers: financial and daily housework burdens, limited social communication and psychological stress.

#### Financial and daily housework burden

Fourteen of the 20 interviewees mentioned high cost of caring for the patients, imposing heavy financial burdens on them. The interviewees were all engaged in substantial amounts of housework. In addition to taking care of the patients, the caregivers also needed to deal with a series of daily tasks and errands to maintain the family. Some of the caregivers wished to hire a housemaid; however, few people were willing to care for people experiencing schizophrenia.

*“In addition to daily living expenses, the cost of visiting a doctor regularly and taking the medication routinely for her (the patient) is also high. She (the patient) once stayed in the mental hospital for 7 months, and ten thousands YUAN (RMB) were paid totally.” (C19).*

*“He (the patient) never does any housework. His mother died, so all the housework depends on me alone. The chores seem endless…”* This was said by a 76-year-old retired father who had taken care of his son for more than 20 years. *(C15).*

*“We once hired a housemaid to take care of her, but she (the patient) distrusted the housemaid extremely because of the fear of being hurt. Thus, it is hard to find a suitable housemaid even with high salary.” (C16).*

#### Limited social communication

Most of the caregivers complained that they had limited social and interpersonal communication opportunities because of accompanying people experiencing schizophrenia and keeping them in their sights.

*“She (the patient) scolded my friend by sending text messages. From then on, my friend never contacted with me. She relies on me so much that I do not have any entertainments.” (C18).*

*“Relatives and friends do not want to bother us after they knew there was a (schizophrenia) patient in our family. The neighbours just greet when we meet together… I am very upset.” (C15).*

#### Psychological stress

Prejudice and discrimination from others with respect to schizophrenia and self-stigma were huge psychological stresses on caregivers, also resulting in social isolation. Most of the caregivers felt the lack of self-security while caring for the patients. Meanwhile, 11 of the caregivers, who were the patients’ parents, were extremely worried about the future life of the patients after they pass away.

*“I do not want to tell anyone (there is a patient in our family). …well… you can feel their attitude…it means your daughter is mad. It is a stigma of the whole family.” (C3).*

*“I live with her now… I feel extremely insecure. She once suddenly broke into my father’s room with a knife at night when my father lived with her.” (C16).*

*“He has to live on himself if we are too old to care for him. We expect that the national policy will be better, and schizophrenia patients could be well managed. We will die in peace.” (C18).*

Fourteen of the 20 caregivers had anxiety or depression, and 7 of them were currently receiving therapy.

*“My eldest son committed suicide due to depression, and my youngest son suffered from schizophrenia. I always cry alone with the thoughts of going to death. Now I am on antidepressants every day.”(C4).*

### Support

In this part, three support subcategories were identified, including financial support, medical support, and information and education support. However, these means of support were all insufficiently available.

#### Financial support

The caregivers expressed that external financial resources including medical insurance and free medication were provided to alleviate family economic pressure at a certain extent.

*“Our total family income is 1200 RMB (≈ 190 U.S. dollars) per month, and we live in a low-rent house. Fortunately, he (the patient) has basic living allowance and 80% of medical expenses can be reimbursed. I am entitled to the ‘old and young’ medical insurance (in Beijing), in which 50% of medical expenses can be reimbursed.” (C20).*

*“We received 2200 YUAN (RMB) subsidies per month from the government. The medications, physical examinations and the insurance were free.” (C1).*

#### Medical support

The interviews showed that medical resources from society and the community alleviated the caregiver’s burdens. For example, the rehabilitation institution as a “respite care” saved private time for caregivers and helped patients to recover and get back to social life; however, the cost was expensive.

*“The rehabilitation activity in rehabilitation institution is helpful. In fact, it is ok as long as he (the patient) can contact with the society. We expect the activity would become routine and the cost of the activity is acceptable.” (C15).*

*“If the patient has a sudden relapse, doctors of CHSC, neighbours, and policemen would help me to prevent his violent behaviour and send him to hospital together.” (C2).*

#### Information and education support

Health education and mutual support groups organized by the community created opportunities for caregivers to communicate with others, increase their knowledge, and alleviated the psychological pressure of caregivers; however, this was not widely accepted by caregivers.

*“CHSC provides health education to schizophrenia caregivers in the community. This is a psychological consult, as well as a way to relieve pressure.” (C12).*

*“It (participating mutual support group) is good, but… I have no time to attend. I have to look after the patient.” (C19).*

### Further needs

Three needs could be identified in this part of the study: more financial support, being respected, and rehabilitation institutions.

#### More financial support

Most of the caregivers hoped that the government could supply more types of free medications and expand health insurance coverage for people experiencing schizophrenia.

*“The free medications alleviated our financial burden for a large extent, but 99% of them were Generation 1 with obvious side effects. We hope that the free-medication directory can extend to Generation 2 medications with fewer side-effects.” (C1).*

*“My daughter has never worked before. Our whole family’s income depends on my husband’s retirement pension… the treatment fee for the patient is expensive. We hope to get more free medical service for patients.”(C3).*

#### Being respected

The interviewees indicated that families with people experiencing schizophrenia are more vulnerable to discrimination in China. They hoped to create a non-discriminatory environment in the entire society. Caregivers want to maintain the reputation, status, rights and health of patients and themselves, but these issues were paid less attention.

*“…Someone calls my son idiot and even beats him sometimes. I hope that (schizophrenia) patients should be fairly treated.”(C4).*

*“Most Chinese still discriminate schizophrenia patients. They would not go to the grocery store where the seller was a* person experiencing schizophrenia*.”(C6).*

#### Rehabilitation institutions

Every interviewee agreed that more affordable rehabilitation institutions should be opened for schizophrenia patients.

*“I think trusteeship institutions should first face the severe patients, who would bring great harm to the family and society. Government should settle down these people well in a place to spend their remaining years in comfort.”(C16).*

*“I heard of Daxing Farming Therapy Base. Patients could do agriculture work or sports and the diet and daily life is taken care of by doctors and nurses. We would like to send patient there, but we can’t afford it.” (C11).*

## Discussion

Schizophrenia has been generally recognized as a public health issue [35]. Family caregivers play crucial roles in taking care of people experiencing schizophrenia in the community. Caregiver preparation and knowledge can significantly affect the long-term outcome of people experiencing schizophrenia in both socioeconomic and clinical terms [36]. Analysing burdens and needs of care from the caregivers’ point of view can consolidate family caregiving support systems effectively and provide a basis for developing rational treatment measures and public health policies.

Unemployment, unmarried/single status, divorce, being middle-aged and low family economic status are associated with an increased risk of schizophrenia [37]. The economic status of families with people experiencing schizophrenia declines over time with a longer duration of illness [38]. The result of our study also showed that most of the people experiencing schizophrenia were unmarried or divorced, unemployed and middle-aged. Furthermore, a low family income and high treatment costs for patients in most of these families result in massive economic pressure. These factors hinder patients from entering into and maintaining normal social and occupational activities, ultimately imposing heavy burdens on those caring for patients. In agreement with earlier findings [17], our study provided strong evidence that psychiatric stigmatization and discrimination prevented patients and their caregivers from attending social activities and resulted in a lack of social communication. The issue of family shame and stigma are prevalent in Chinese culture and result in even more difficult problems for these caregivers [16]. To avoid feeling trapped and embarrassed, families with people experiencing schizophrenia are often reluctant to seek help and may underuse mental health care services [11], further exacerbating the financial, physical and psychological burdens on the caregivers.

Although most of the patients in our study were in a stable or relatively stable status, the caregivers still expressed concerns that the patients brought potential danger to the caregivers’ security, consistent with findings of a previous study. That epidemiological work [35] indicated that 68.8% patients exhibited threatening behaviours, and 5.4% patients reportedly made threats to others’ property and personal security. Violence and self-harm behaviours of people experiencing schizophrenia cause great public health concerns and challenges [35].

In our study, most of the caregivers were the parents of the patients, and they undertook additional burdens, including psychological stress and substantial amounts of housework, when caring for the patients. A study confirmed that parental caregivers were reported to have higher degrees of burden than sibling caregivers [17]. The parents residing with patients were more likely to experience high levels of distress [39].

Understanding the sources of caregiver burden has a significant impact on improving family intervention programmes, patient outcomes and the caregivers’ well-being. To a certain extent, the available services, resources, and support for patients and their family caregivers can relieve the burdens on family members taking care of the patients [11]. When attention is paid to the prevention and control of the disease, corresponding interventions and destigmatization programmes for caregivers should also be provided. Emotional stability in caregivers may help maintain the quality of care they provide to their patients and may reduce medical expenses by decreasing rehospitalization rates [40]. Many measures have been implemented to combat mental diseases. WHO issued the “Mental Health Action Plan 2013–2020” in May 2013 with the following objectives: strengthen effective leadership and governance for mental health; provide comprehensive, integrated and responsive mental health and social care services in community-based settings; implement strategies for promotion and prevention in mental health; and strengthen information systems, evidence and research for mental health [1].

The “National Standards for Basic Public Health Services” [41] were disseminated in 2011 and updated in 2017 by the National Health and Family Planning Commission of the PRC (now renamed the National Health Commission of the PRC). In the Standards, medical professionals at CHSCs are required to provide the following free services to patients with severe mental disorders: electronic health record establishment; follow-up and evaluation of the patients’ conditions four times a year; treatment of patients with various conditions with pharmacological interventions; symptom and illness management; and physical examination once a year. When following-up patients, medical professionals also provide psychological support and help for caregivers. In 2012, the National Health and Family Planning Commission (now renamed the National Health Commission of the PRC) issued the “Training Regulation for Prevention and Treatment of Severe Mental Illness”, aiming to improve the basic knowledge and skills of mental health professionals for the prevention and treatment of mental illness and enhance the service capacity of psychiatric specialist institutions [42].

Although the government provides multi-channel resources for people experiencing schizophrenia and their families, these resources remain limited, and utilization rates of mental health services or the proportion of help-seek behaviours remain low in China [5]. It is evident that the majority of the patients with severe mental disorders did not receive professional therapy or were not treated very well [35]. The main reasons for this lack of treatment are summarized as follows: (1) The workforce of rehabilitation and social care professionals in China is insufficient [43]. Psychoeducation provided by psychiatrists and psychiatric nurses is rather confined to the clinical aspects of mental illness [44], and the development of rehabilitation and recovery concepts is hindered [8]. (2) Funding and other support from national, local and social institutions and organizations for mental illness are limited. Meanwhile, complex application procedures and limited quota allocations limit access to various types of support [45]. (3) Stigma and discrimination from the public and even mental health professionals [46], low mental health knowledge and perceived need, and low insurance coverage with high treatment fees created barriers to mental health service utilization [35]. The caregivers in our study also complained of a low percentage of subjects treated for schizophrenia due to high treatment fees and stigma. (4) Current support is primarily supplied for people experiencing mental diseases. Support for caregivers is extremely lacking and cannot meet the needs of caregivers. The mutual support groups and psychological counselling provided for caregivers are often considered superficial and impractical [47].

Based on these results, we have a number of policy and service development suggestions. First, further broadening the coverage of healthcare insurance for severe mental diseases and providing more disability income support and accessible financial protection for patients with severe mental diseases are important countermeasures to support more patients seeking medical health services [5]. Income support and financial protections include extending free medication directories; increasing the proportion of reimbursement for hospitalization and rehabilitation; simplifying the procedures of applications and acquisition of supports; and increasing the amount of support provided. Second, the government should provide more sustainable resources to facilitate integrated individualized care from specialist institutions to communities [12]. Our study explored the views of the caregivers towards the needs for community psychiatric rehabilitation, in agreement with the findings of a previous study [8]. Strengthening the existing primary mental healthcare system may be another important measure for treating mental diseases in addition to expanding medical insurance coverage. Community-based rehabilitation services are generally lower in cost than hospitalization and allow for better geographical access [48]. Community-based psychiatric rehabilitation can promote quality of life for both persons with mental illness and their families by achieving social inclusion via the joint efforts of stakeholders including the patients and their caregivers, professionals, service providers and the government (WHO, 2004). Policy makers should allocate more resources for community care, train more community care workers and make referral process effective [8]. It is urgent to establish and train a high-quality multidisciplinary community team, consisting of psychiatrists, psychiatric nurses, GPs, public health professionals, psychotherapists, social workers and caregivers, for the rehabilitation of people experiencing schizophrenia. Third, anti-discrimination policies and mental health public education activities through social media and information packages should be implemented by the government to protect the patients and their caregivers from explicit discrimination in the public arena and to provide them with equal opportunities to those of other citizens to participate in various social activities [46]. Fourth, to improve the mental health services, involvement of the families in policy making and service planning becomes essential. Clinicians, mental health practitioners and policy planners should better assist and utilize family members in the management and delivery of care for people experiencing schizophrenia [36]. The health professionals should recognize caregivers as significant players in the establishment and maintenance of the working alliance for people experiencing schizophrenia [36] and should train and tailor the support to caregivers in order to facilitate their engagement in the recovery process of patients [8]. Furthermore, family caregivers should be involved as active members of the health care team taking care of the patients [11, 12]. Meanwhile, medical professionals should assess the emotional status of caregivers who are considered “potential patients”, provide services on stress management, and encourage their participation in support groups to reduce the caregiver discomfort and enhance their well-being. Furthermore, affordable or free temporary day rehabilitation institutions for patients should be constructed so that caregivers can have time to participate in support groups and counselling. Last but not least, supported employment and social skills training for patients should be strengthened. Mental health professionals and policy makers should allocate more sufficient resources to implement strategies identifying high-risk populations, provide vocational training and supported employment for patients seeking jobs [49, 50].

This is the first in-depth study exploring insights regarding burdens, support and further needs from the perspective of the caregivers of people experiencing schizophrenia in the community context in mainland China. One limitation is the relatively small sample size in Beijing. Future research is recommended to explore important factors influencing their attitudes and decisions concerning caring for people experiencing schizophrenia on a larger scale, including suburban areas.

## Conclusions

This qualitative study provided insight from family caregivers of people experiencing schizophrenia regarding care burdens and support. Family caregivers suffer from heavy physical and psychological burdens, including financial and daily housework burdens, limited social communication, and psychological stress. The current support provided to them is insufficient. More financial support, more organization services and improved public attitudes should be provided to people experiencing schizophrenia and their caregivers.

## Abbreviations

CHSC: Community health service centres
SNQ: Social Network Questionnaire
WHO: World Health Organization
